# Discovery of J Chain in African Lungfish (*Protopterus dolloi*, Sarcopterygii) Using High Throughput Transcriptome Sequencing: Implications in Mucosal Immunity

**DOI:** 10.1371/journal.pone.0070650

**Published:** 2013-08-14

**Authors:** Luca Tacchi, Erin Larragoite, Irene Salinas

**Affiliations:** Center for Evolutionary and Theoretical Immunology, Department of Biology, University of New Mexico, Albuquerque, New Mexico, United States of America; INRA, France

## Abstract

J chain is a small polypeptide responsible for immunoglobulin (Ig) polymerization and transport of Igs across mucosal surfaces in higher vertebrates. We identified a J chain in dipnoid fish, the African lungfish (*Protopterus dolloi*) by high throughput sequencing of the transcriptome. *P. dolloi* J chain is 161 aa long and contains six of the eight Cys residues present in mammalian J chain. Phylogenetic studies place the lungfish J chain closer to tetrapod J chain than to the coelacanth or nurse shark sequences. J chain expression occurs in all *P. dolloi* immune tissues examined and it increases in the gut and kidney in response to an experimental bacterial infection. Double fluorescent in-situ hybridization shows that 88.5% of IgM^+^ cells in the gut co-express J chain, a significantly higher percentage than in the pre-pyloric spleen. Importantly, J chain expression is not restricted to the B-cell compartment since gut epithelial cells also express J chain. These results improve our current view of J chain from a phylogenetic perspective.

## Introduction

J chain is a unique 15 KDa polypeptide that is incorporated in the polymeric immunoglobulins such as IgM and IgA. Initial characterizations of J chain in humans and mice revealed a high degree of conservation in this molecule, with 8 Cys residues and a high abundance of acidic residues [1]. Two of the J chain Cys residues are responsible for the disulphide bridges that bind J chain to Ig H chain. Thus, mammalian J chain and the Cμ4 and Cα3 domains in IgM and IgA are the most conserved elements in the immunoglobulin system indicating that the structural requirements for Ig polymerization have imposed powerful selective constraints [1]. Although IgA^+^ and IgM^+^ B cells and plasma cells typically express J chain, in the absence of IgA, IgG^+^ and IgD^+^ cells are capable of expressing high levels of J chain [2], [3]. Additionally, J chain^+^ IgD and IgG producing cells occur in tissues with glandular elements in mammals [4].

Beyond its function in Ig polymerization, J chain is involved in the transport of Ig across epithelial surfaces by assisting their binding with the poly Ig receptor (pIgR) [5]–[7]. Due to the central role of J chain at mucosal surfaces, there are important differences in the expression of J chain by different B cell subsets. For instance, most IgA and IgM producing cells in normal intestinal and nasal mucosa show 100% J chain positivity, whereas B cells from peripheral lymph nodes have a low J chain profile expression [3].

J chain has been identified in a number of non-mammalian vertebrate species including birds, reptiles, amphibians and cartilaginous fish [8]–[12] whereas it does not appear to be present in teleosts [13]. Comparative studies of J chain in lower vertebrates have shown that the 8 Cys conservation is not present in *Xenopus* and nurse shark. Thus, some functional properties of the J chain including its ability to polymerize Igs were gained in later vertebrates [11], [14]. Additionally, J chain has been identified in invertebrate species that lack Ig genes [15] which first prompted the question of whether J chain may possess other Ig-independent defense functions. However, the presence of J chain in invertebrates is to date still disputed [16], [17]. Moreover, J chain is not restricted to the B cell compartment in mammals, since a population of dendritic cells expresses J chain protein [18]. Thus, comparative studies on J chain suggest a possible enigmatic function for this molecule other than the Ig polymerization and mucosal transport roles first described in mammals [17].

Dipnoi fish, such as the African lungfishes, are sarcopterygian or lobed-fin fishes with a very interesting phylogenetic position. Based on molecular systematic studies, lungfish represent the closest ancestor of tetrapods [19]–[21]. Dipnoi fish express Igs and it has been demonstrated that African and Australian lungfish possess 19S and 5.8S serum Igs [22], [23]. Despite of this, little is known regarding their function, polymerization status and tissue specificity. A previous study on African lungfish *Protopterus aethiopicus* revealed the presence of IgM and IgW (IgD) molecules [24].

IgM is the only class of Ig conserved in all the vertebrates species and its heavy chain consists of one V domain, a DJ region, and four CH domains [25]. However, due to an alternative splicing pathway, teleost membrane IgM lacks the CH4 domain [25]. An orthologous of cartilaginous fish IgW was found in the African lungfish [24]. Lungfish IgW can have either 7 or 2 CH domains. These short and long IgW forms may derive by alternative splicing or they are a product of a recent gene duplication [24]. Lungfishes share a common ancestor with sharks 460 million years ago (MYA), phylogenetic analysis of cartilaginous fish and lungfish IgM and IgW demonstrated they form two major ancient gene groups. Since molecular phylogenetic analyses determined that the cartilaginous fish (Chondrichthyes) separated prior to the divergence of bony fishes (Osteichthyes, both lobe-finned and ray-finned) from the other jawed vertebrates the discovery of IgW in the lungfish suggested that it was present in the common ancestor of bony and cartilaginous fishes 460 MYA.

Despite having the two most ancestral Ig isotypes of all vertebrates (IgM and IgW) very little is known about the immune system of sarcopterygian fish and whether or not this vertebrate group has a dedicated mucosal immune system. Furthermore, lungfishes have a unique intestine structure characterized by a spiral valve (similar to that of sharks) with a pyloric fold separating the foregut from the midgut [26]. A pre-pyloric groove is present and it contains a pre-pyloric spleen while a separate post-pyloric spleen is situated in the free margin of the spiral valve [26].

To date, J chain had not been described in Dipnoi or any sarcopterygian fish species and the present study reports for the first time the J chain of sarcopterygian fish, the Nigerian spotted lungfish (*Protopterus dolloi*). We provide amino acid sequence comparisons with other vertebrate J chains and give insights into the function of J chain in lungfish by investigating its expression in central and mucosal tissues in an infection model as well as the cellular co-expression of J chain with IgM and IgW. In addition, we show that J chain is not confined to the B-cell compartment but is also expressed in non-lymphoid cells like the gut epithelial cells.

## Materials and Methods

### Fish

Juvenile Nigerian spotted lungfish (*P. dolloi*) (9–12 inch total length) were obtained from Segrest farms and maintained in 10-gallon aquarium tanks at a constant temperature of 27°C. 10% of the tank water was exchanged daily. Fish were fed three times a week frozen earthworms. Feeding was terminated 48 h before sacrifice. Fish were acclimated to the laboratory conditions for at least 2 wk before being used in experiments.

### Ethics statement

All animal studies were reviewed and approved by the Institutional Animal Care and Use Committee (IACUC) at the University of New Mexico, protocol number 11-100744-MCC.

### Identification of P. dolloi and coelacanth J chain sequences and cloning of P. dolloi J chain, IgM and IgW

Initial identification of a J chain molecule in *P. dolloi* was done by 454 pyrosequencing of the transcriptome of the pre-pyloric spleen of a *P. dolloi* individual. Total RNA was isolated from pre-pyloric spleen tissue, and mRNA was isolated from total RNA with the MPG® mRNA Purification Kit (PureBiotech). cDNA was made using the cDNA Synthesis System Kit with random primers (Roche). A cDNA Rapid library was constructed with the GS FLX Titanium Rapid Library Preparation Kit. Emulsion-based (em) PCR amplification of the DNA library was carried out with the GS FLX Titanium LV emPCR Lib-L Kit. Pyrosequencing was conducted using a GS FLX Titanium Sequencing Kit XL+ in a GS FLX+ System. All reagents and protocols used were from Roche 454 Life Sciences, USA. A total of 1 million reads were generated with a mean length of 425 bp. 136,857 of them were contigs and singletons. The J chain sequences available in GenBank (accession no. AAC05636.2, AAH06026.2, AAO24897.2) were used to find a homologous J chain sequence in the *P. dolloi* 454 read database using BLAST. A partial sequence of 381 bp was identified as J chain gene. The sequence lacked the 3′ and 5′ UTR and 3′ RACE PCR was performed using specific primers (Table S1 in File S1) to obtain the full ORF as described previously [27]. The 5′ RACE PCR was performed using 5′/3′ RACE kit (2nd Generation, Roche) according to the manufacturer's instructions. All PCR products were ligated into pGEMTeasy (Promega) vector and cloned into TAM1 competent cells (Active Motif). Plasmid DNA was isolated from positive colonies using the QIAprep Miniprep kit (Qiagen) and sequenced with an ABI 7000 sequencer (Applied Biosystems) at the Molecular Biology Core Facility of the Biology Department, University of New Mexico. The nucleotide sequence submitted to GenBank is available with the accession no. JX999962. The J chain sequence of *Latimeria chalumnae* (accession n° ENSLACP00000023629) was identified by data mining using genomic sequences for orthologous genes within the Ensembl Genome Browser (http://ensembl.org/).

Additionally, we identified two IgH sequences using available *P. aethiopicus* IgM and IgW sequences (accession no. AF437735.1 and AF437726.1). Partial sequences found in the *P. dolloi* 454 database were further cloned as explained above using specific primers (Table S1 in File S1). Nucleotide sequences submitted to GenBank are available with the accession no. JX999963 (*P. dolloi* IgM) and JX999964 (*P. dolloi* IgW).

### Sequence analysis

Prediction of the open reading frame (ORF) was performed with the programs BLAST (Altschul et al., 1990) and the ExPASy proteomics server (ca.expasy.org/). Multiple sequence alignments were generated using CLUSTAL W (http://align.genome.jp/) [28]. Phylogenetic tree was constructed from generated alignments using the Neighbour-Joining (NJ) method within the software MEGA 4 [29] and was bootstrapped 1000 times. To obtain the identity of the sequences the software MatGat 2.02 [30] was used.

### Experimental infection and tissue samples

**Figure 1:**
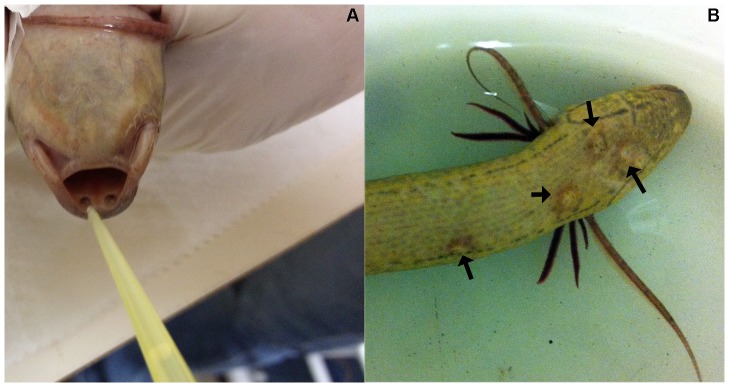
Experimental bacterial infection model in *P. dolloi*. A) Delivery of *E. ictaluri* to *P. dolloi* intranasally. B) Skin ulcers (black arrows) produced by *E. ictaluri* in an infected *P. dolloi* individual. No ulcers were observed in control specimens.

*P. dolloi* were experimental infected with the Gram negative enterobacterium *Edwardsiella ictaluri* strain J100 [31]. *E. ictaluri* is the causative agent of enteric septicaemia in channel catfish and grows optimally at 25–30°C the rearing temperature of *P. dolloi*. Briefly, *E. ictaluri* was grown in Bacto-Brain Heart Infusion broth at 27°C for 48 h. An OD_600_ of 1 corresponded to 2×10^8^ cfu/ml as assessed by plate counts. Bacterial cultures were washed three times in PBS and 100 µl of a 2×10^7^ cfu/ml suspension were delivered into the olfactory canal of *P. dolloi* (Figure 1A). Since the olfactory organ is connected to the upper roof of the oral cavity in *Protopterus spp.*, the bacterial suspension accessed the upper gastrointestinal tract. Twenty-one days after the first immunization, fish received a second dose following the same protocol. Fish were sacrificed 10 days after the secondary immunization. No fish died due to the experimental infection but two individuals showed red and white lesions in the skin and head (Figure 1B). Reddening inside and around the buccal cavity was often observed in infected specimens even in the absence of lesions. Both signs are typically associated with *E. ictaluri* infections in teleost fish [32], [33]. Control and infected specimens were euthanized by a lethal dose of MS-222 diluted in water. After bleeding from the caudal vein, pre-pyloric spleen, post-pyloric spleen, kidney, skin, gills and gut tissues were collected and placed in RNAlater (Invitrogen) for total RNA extraction.

### Laser capture microdissection of gut epithelial cells

The posterior gut of two control *P. dolloi* specimens was snap frozen in OCT (TissueTek). Serial 5 µm cryosections were obtained. One of the sections was stained with H&E and the rest were used for dissection of epithelial cells. Gut epithelial cells were isolated from cryosections of control *P. dolloi* gut using an ArcturusXT Laser Capture Microdissection (LCM) microscope (Applied Biosystems). Approximately 5,000 cells from two different individuals were captured and combined. Total RNA was extracted from LCM epithelial cells using Arcturus PicoPure RNA Isolation Kit (Applied Biosystems) following manufacturer's instructions. The RNA was stored at −80°C until required for cDNA synthesis. cDNA synthesis was performed using 500 ng of total RNA, which was denatured (65°C, 5 min) in the presence of 1 µl of oligo-dT17, 1 µl dNTP (deoxynucleoside triphosphate mix 10 mM each (Promega) and RNA/DNA free water (Sigma) in a volume of 13 µl. The RNA was then cooled on ice, and synthesis carried out using 1 µl Superscript III enzyme reverse transcriptase (Invitrogen) in the presence of 5 µl of 5× first strand buffer, 1 µl 0.1 M DTT, made up to a final volume of 25 µl with water, and incubated at 55°C for 1 h. The resultant cDNA was diluted to a final volume of 50 µl in RNA/DNA free water (Sigma) and stored at −20°C. The cDNA was used in RT-PCR using specific J chain, IgM, IgW and cytokeratin-8 (CK-8) primers (Table S1 in File S1). The J chain PCR product from the LCM gut epithelial cells was cloned as described above to confirm the sequence.

### Quantitative real-time PCR (qPCR) analyses of P. dolloi J chain expression

qPCR was used to determine the abundance of J chain transcripts in tissues of control and infected *P. dolloi*. The qPCRs were performed in triplicate and each contained 3 µl of a diluted cDNA template (4 ng of total RNA equivalents), 12.5 µl of Power SYBR Green PCR master mix (2×; Applied Biosystems), and 150 nM forward and reverse primers in a 25 µl reaction volume. The amplification profile consisted of an initial denaturation step at 95°C for 10 min, and then 30 cycles of 95°C for 15 s and 60°C for 1 min followed by melting (dissociation stage) from 72°C to 95°C in an ABI Prism 7000 (Applied Biosystems) sequence detection system. A negative control (no template) reaction was also performed for each primer pair. A sample from the serial dilution was run on a 2% agarose gel and stained with RedGel Nucleic Acid Stain (Biotium) and viewed under UV light to confirm a band of the correct size was amplified. A melting curve for each PCR was determined by reading fluorescence at every degree between 72°C and 95°C to ensure only a single product had been amplified. *P. dolloi* elongation factor EF-1α (KC579465) was used as control gene for normalization of expression.

The relative expression level of the genes was determined using the Pfaffl method [34]. Efficiency of the amplification was determined for each primer pair using serial 10 fold dilutions of pooled cDNA, performed on the same plate as the experimental samples. The efficiency was calculated as E = 10 (−1/s) where s is the slope generated from the serial dilutions, when Log dilution is plotted against ΔCT (threshold cycle number). Primers were designed to have a Tm of 55°C, and where possible, to cross an exon-exon junction to avoid amplification of genomic DNA. Exon-intron junction sites were determined by comparing the *P. dolloi* cDNA with genomic sequence for orthologous genes from other vertebrates obtained from Ensembl (http://ensembl.org/).

### Fluorescence in situ hybridization (FISH)

The pre-pyloric spleen and gut of three control *P. dolloi* specimens was snap frozen using OCT (TissueTek). Cryosections (5 µm-thick) form each cryoblock were obtained and used for FISH staining. Detection of J chain, IgM and IgW transcripts on *P. dolloi* lymphoid tissues was conducted by FISH using Stellaris custom probes (Biosearch Technologies, Inc.). Stellaris FISH probe sets consist of multiple singly labeled oligonucleotides (generally 30–48) designed to hybridize along targeted RNA transcripts. The potential for false positives is low because the signal is only detectable when tens of probes are bound [35], therefore a negative control is not required. Using this technology, each individual dot corresponds to one target RNA transcript. The entire J chain nucleotide sequence was used to design the probe, whereas the IgM and IgW probes lack the nucleotide sequences for the IgV domain. Single J chain stains as well as double J chain and IgM or J chain and IgW stains were performed on cryosections from pre-pyloric spleen and gut (n = 3). Four cryosections were stained per sample and 20 images (×60 magnification) were acquired under a Nikon Ti epifluorescence microscope. Two independent readers evaluated every slide in a blind fashion to avoid biased counts. Images were analyzed using the Advanced Research NIS Elements software (Nikon).

### Statistical analysis

Data is presented as mean ± standard error. qPCR measurements were analyzed by t-test by comparing values with either the EF-1 control or the non-infected control. One-way ANOVA analysis followed by Tukey's posthoc test was used to identify expression differences among tissues. Two-way ANOVA was used to determine significant differences in the double FISH studies. p-values<0.05 were considered significant.

## Results

### Sequence analysis of lungfish J chain

**Figure 2:**
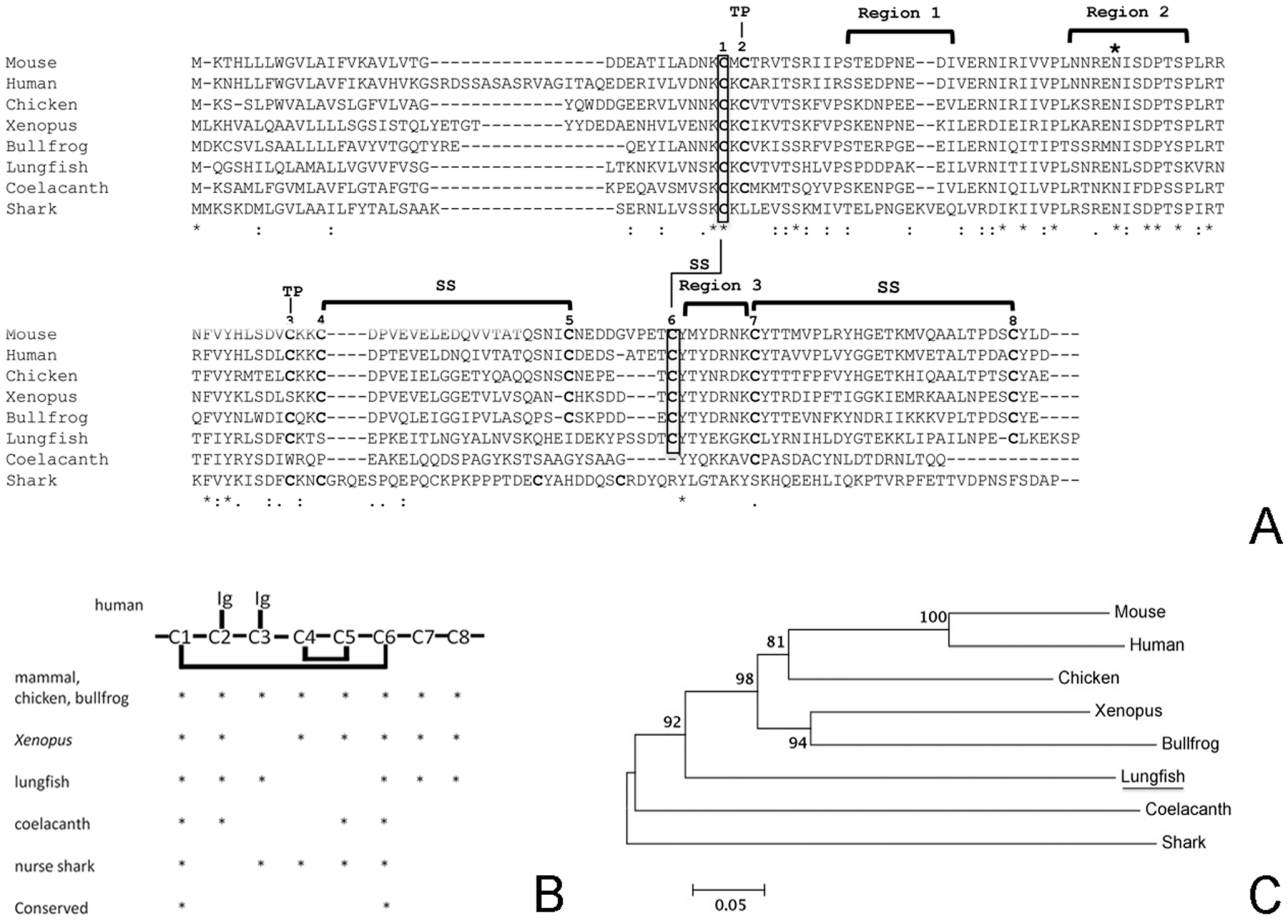
Lungfish J chain molecular analysis. A) Alignment of the J chain amino acid sequences of lungfish (JX999962), mouse (AAH06026), human (ABI63362), chicken (BAB83927), *Xenopus* (AAC05636), bullfrog (ACO52584), coelacanth (ENSLACP00000023629) and shark (AAO24897). “SS” indicates the interchain disulfide bridges and “TP” marks the Cys paired with the secretory tail of IgA or IgM. The conserved N-linked glycosilation site is indicated by “+”. Numbers (1–8) indicate the Cys residues in human sequences. “*” Indicates identical amino acids between all sequences, whilst “.” and “:” show conservative substitutions. Regions 1–3 were identified as in Braathen et al (2007) B) Conservation of Cys residues between lungfish and other vertebrates. C) Phylogenetic tree showing the evolutionary relationship of J chain molecules in vertebrates. The tree was constructed using the Neighbour-Joining method in MEGA 4 and was bootstrapped 10,000 times. GenBank accession numbers are as follows: lungfish (JX999962), mouse (AAH06026), human (ABI63362), chicken (BAB83927), *Xenopus* (AAC05636), bullfrog (ACO52584), coelacanth (ENSLACP00000023629) and shark (AAO24897).

A J chain sequence (JX999962) was identified and cloned in *P.dolloi*. The sequence is 819 bp, and contains a 5′-UTR of 48 bp, an open reading frame of 483 bp encoding an ORF of 161 amino acids and a 3′-UTR of 288 bp. The alignment of the deduced amino acid sequences of *P. dolloi*, coelacanth and other available vertebrate J chain sequences is presented in Figure 2A. In terms of conservation of Cys residues, out of the 8 Cys present in mammalian J chain (numbered Cys1-8), *P. dolloi* J chain has 6, with Cys 4 and 5 missing. This implies that lungfish J chains forms one less intramolecule disulphide bond than that of mammals, birds and amphibians. Cys 2 and Cys 3 form disulphide bonds with Cα and Cμ. Cys 3, located in residue 70, is missing in *Xenopus* and shark. Interestingly, lungfish J chain has had both Cys 2 and Cys 3 conserved indicating that double disulphide bonds may link the J chain to form polymeric IgM or IgW. Additionally, Cys 6 is not conserved in the nurse shark but it is present in *P. dolloi*. In the coelacanth, only 4 of the 8 Cys residues are found as shown in Figure 2. Overall, 3 Cys are conserved from coelacanth to humans and only 2 Cys are conserved from nurse shark to humans (Figure 2B). The important N-glycosylation site at position 50 is also conserved in *P. dolloi* but not in *L. chalumnae*. Lungfish J chain residue 84 is a Leu, similar to all tetrapods, however the nurse shark J chain has an additional Cys residue at this position. In the coelacanth, in turn, residue 84 is a Glu, therefore losing hydrophobicity.

As shown in Table S2 in File S1, the deduced lungfish J chain amino acid sequence shows amino acid identities of 44/45% to chicken J chain, 40/41% to *Xenopus* J chain, 37/38% to mouse J chain, 36% to bullfrog J chain, 36/37% to human J chain and 31/32% to coelacanth and to shark J chains. Thus, lungfish J chain is 6% more similar to mammalian J chain than to coelacanth and shark J chains. Importantly, the lungfish J chain is 13% more similar to the chicken molecule than to the coelacanth and shark molecules. A comparison of J chain sequences from *Xenopus* to humans revealed that two regions are conserved in all tetrapods (Figure 2A). These are located between residues 46 and 57 (region 2) and 131–135 (region 3). Region 2 is believed to be essential for the interaction with pIgR [36], whereas region 3 interacts with the secretory component (SC). The current study adds important information to the evolution of these two regions. *P. dolloi* J chain displays an intermediate degree of similarity between nurse shark and tetrapods. The core of region 2 has an Asn-Ile-Ser-Asp-Pro conserved motif in all vertebrates studied to date. In lungfish, we found Asn-Leu-Ser-Asp-Pro in this region, but the change of Ile by Leu may not alter J chain function since both are hydrophobic residues. Similarly, an Arg residue characterizes the center of region 3 from *Xenopus* to humans but is absent in shark. Lungfish do not have Arg but Lys in this position. Since both amino acids are hydrophilic and positively charged, this substitution could be synonymous and have no functional consequences with respect to the interaction with pIgR and generation of secretory Igs. In contrast, the divergence between coelacanth and tetrapod J chain regions 2 and 3 was much higher (Table S1 in File S1). Only 33/34% and 28/29% of each region, respectively, are conserved in the coelacanth, a lower percentage than in the case of the nurse shark.

### Lungfish J chain is most closely related to the J chain of tetrapod species

The phylogenetic tree of all vertebrate J chain sequences is shown in Figure 2C. Despite the greater sequence similarity between lungfish and chicken J chains, the phylogenetic tree composite shows that all tetrapod J chains cluster together. The lungfish J chain clusters with the tetrapod group. Lungfish J chain is about 31% similar to both coelacanth and shark J chain sequences. However, in the phylogenetic tree, coelacanth J chain appears closer related to the lungfish and tetrapod cluster, with the shark J chain sequence forming a different clade.

### J chain is predominantly expressed in mucosal lymphoid tissue in lungfish

**Figure 3:**
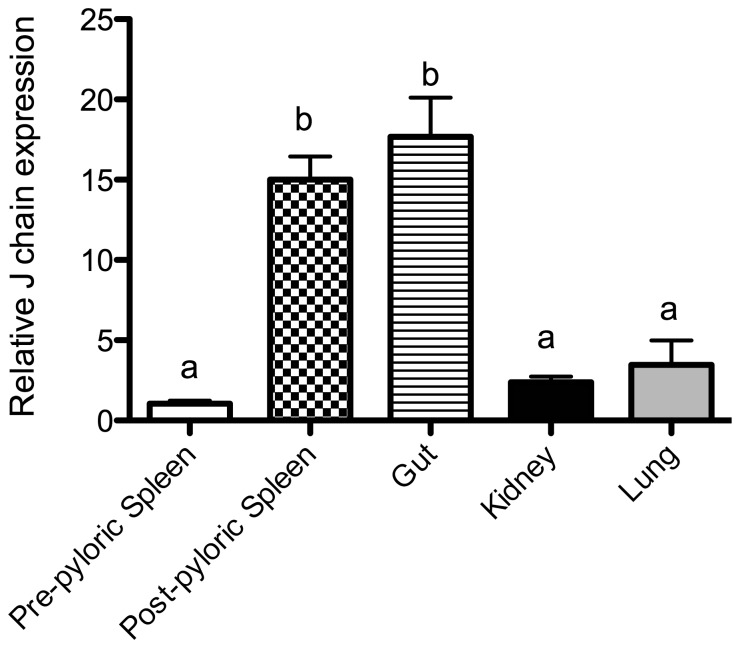
Tissue distribution of J chain expression in control Nigerian spotted lungfish (*P. dolloi*) (n = 6). The relative expression of each gene was normalized first with the house keeping genes, and then divided by the average expression level in the pre-pyloric spleen, which had the lowest value. Bars represent means ± standard error of four fish. Different letters represent statistically significant groups after Tukey's test (p<0.05).

To evaluate the tissue specific expression of J chain in *P. dolloi* we measured by qPCR J chain expression relative to the house keeping EF-1α (Figure 3). The highest expression levels of J chain transcripts were recorded in the gut and post-pyloric spleen, followed by the lung, kidney and pre-pyloric spleen. The expression levels in the gut were ≈5-fold, 7-fold and 17-fold higher than in the lung, kidney and pre-pyloric spleen respectively.

### The majority of IgM^+^ cells co-express J chain in the gut

**Figure 4:**
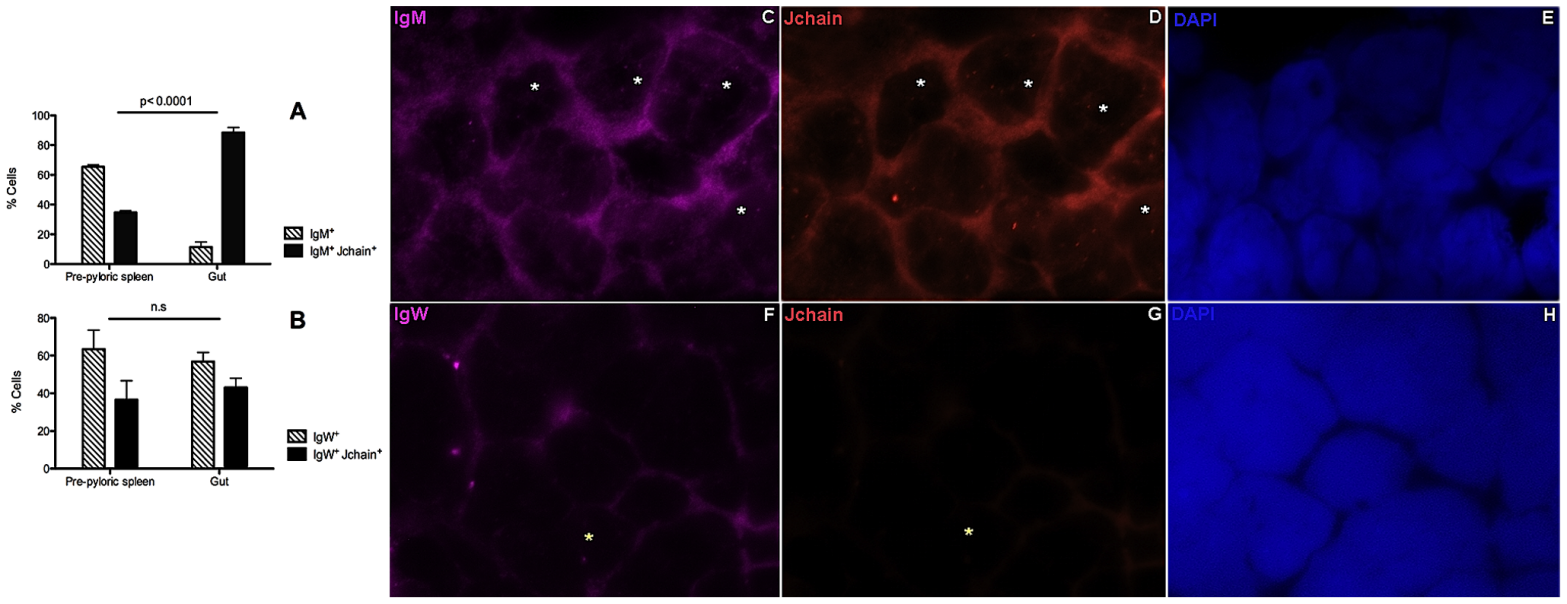
Expression of J chain by B cells of Nigerian spotted lungfish (*P. dolloi*). A) Mean percentage of IgM^+^ and IgM^+^ J chain^+^ B cells and B) Mean percentage of IgW^+^ and IgW^+^ J chain^+^ B cells in the pre-pyloric spleen and gut of control *P. dolloi* (n = 3) studied by double FISH staining. Cells were counted from 20 different fields (×60). Differences were statistically significant when p<0.05. n.s: non-significant differences. C–E) Examples of representative FISH staining showing IgM^+^ J chain^+^ lymphocytes (white asterisks) from a gut cryosection. C) Cy5-IgM FISH probe D) Cy3-Jchain FISH probe E) DAPI nuclear stain. F–H) Examples of representative FISH staining showing a IgW^+^ J chain^−^ lymphocyte (yellow asterisk) from a pre-pyloric spleen cryosection. F) Cy5-IgW FISH probe G) Cy3-Jchain FISH probe H) DAPI nuclear stain.

In order to investigate if *P. dolloi* IgM^+^ and IgW^+^ B cells co-express J chain, we studied the presence of IgM, IgW and J chain transcripts in the pre-pyloric spleen and gut of control *P. dolloi* by FISH. Single staining showed that J chain^+^ cells were more abundant in the gut than in the pre-pyloric spleen, supporting our qPCR tissue distribution results. J chain transcripts were found in cells with lymphocyte morphology. When double staining was conducted, we found that 88.5% of the IgM^+^ cells in the gut were also J chain^+^. In the pre-pyloric spleen, in turn, only 35% of the IgM^+^ cells (p<0.0001) co-expressed J chain (Figure 4A). In the case of IgW, around 50% of the IgW^+^ cells were J chain^+^, a similar percentage to that found in the pre-pyloric spleen (Figure 4B). A representative example of a double IgM^+^ J chain^+^ lymphocyte from the gut is shown in Figure 4C–E and a representative IgW^+^ J chain^−^ lymphocyte is shown in Figure 4F–H. The nucleus to cytoplasm ratio of a typical *P. dolloi* gut lymphocyte is shown in Figure S1A, by overlaying the DAPI stain with a differential interference contrast (DIC) image of the same field.

### Lungfish J chain expression in response to in vivo enterobacterial infection

**Figure 5:**
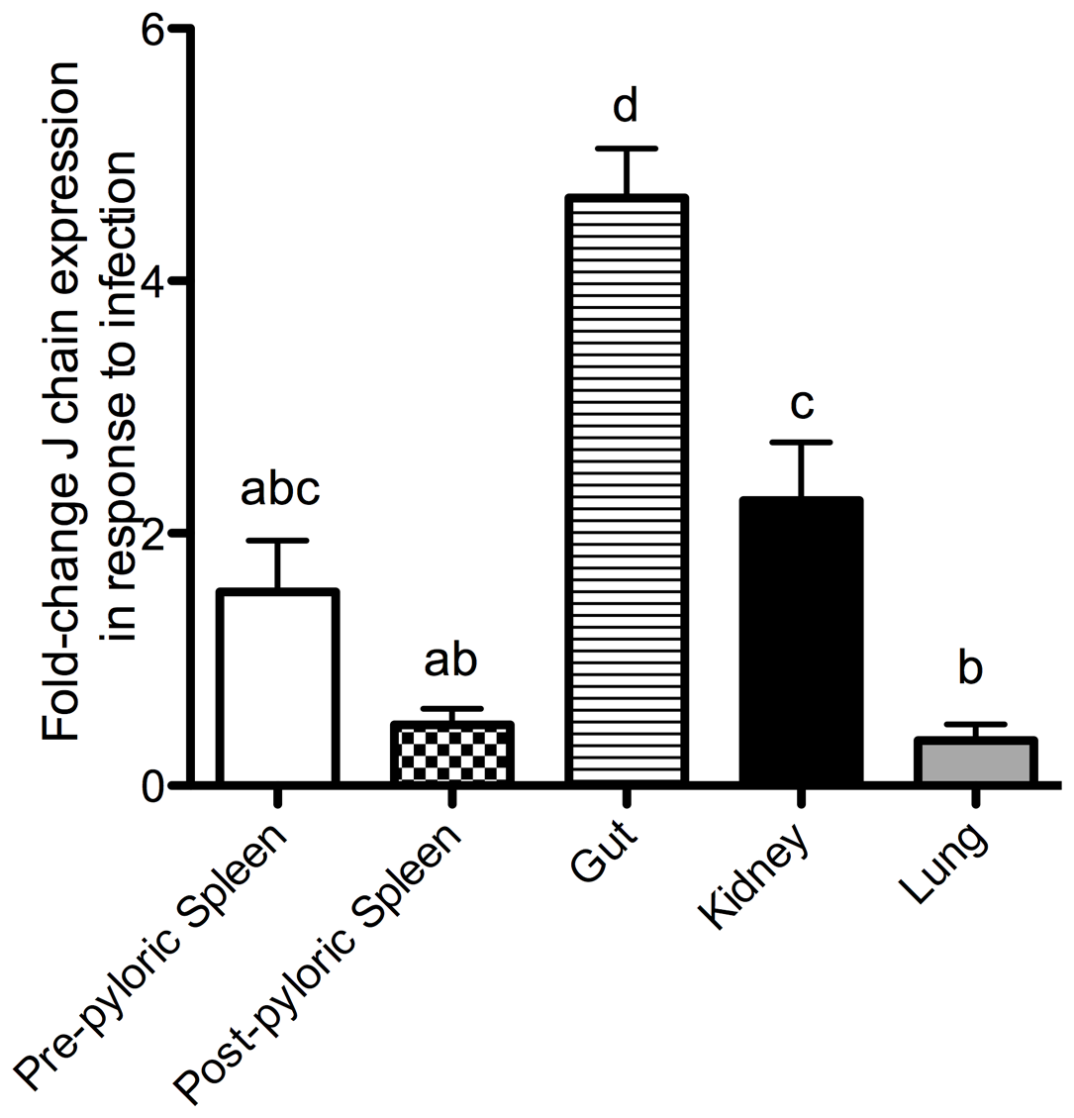
Fold change of J chain expression in pre-pyloric spleen, post-pyloric spleen, gut, kidney and lung of Nigerian spotted lungfish infected with *Edwardsiella ictaluri* compared to samples from PBS mock control fish. Bars represent means ± standard error of four fish. Different letters represent statistically significant groups after Tukey's test (p<0.05).

The expression of J chain transcripts in response to *E. ictaluri* oral infection in different tissues is shown in Figure 5. T-test revealed that J chain expression was significantly up-regulated in the gut (4.6-fold) and kidney (2.25-fold) of *P. dolloi* 10 days after secondary immunization. In contrast, J chain expression was not significantly changed in the pre-pyloric spleen. Down-regulation of J chain expression was significant in the post-pyloric spleen and lung (0.48 and 0.35-fold, respectively). One-way ANOVA and Tukey's posthoc test show that the up-regulation observed in the gut was significantly different to that of any other tissues studied.

### Lungfish gut epithelial cells also express J chain

**Figure 6:**
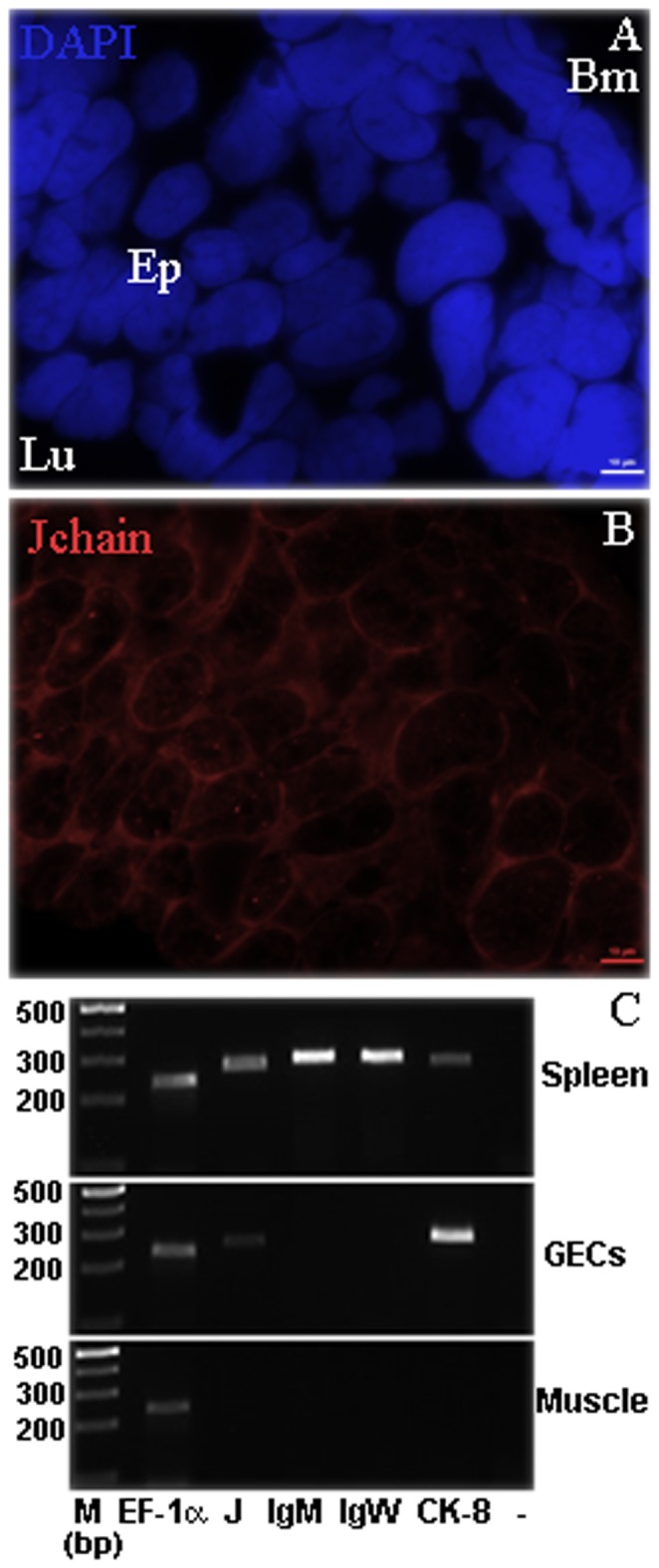
*P. dolloi* gut epithelial cells (GEC) express J chain. A) P. *dolloi* gut cryosection stained with the nuclear stain DAPI (blue) showing the characteristic nuclear morphology of epithelial cells B) *P. dolloi* gut cryosection stained with Cy-3 J chain Stellaris FISH probe. Epithelial cells contained numerous J chain transcripts (dots). Ep: epithelial cells, Lu: lumen, Bm: basal membrane C) Gut epithelial cells were dissected from control *P. dolloi* gut cryosections using LCM. Total RNA from *P. dolloi* pre-pyloric spleen, LCM gut epithelial cells (GEC) and muscle was extracted and used as template for RT-PCR using J chain (J), IgM, IgW and cytokeratin 8 (CK-8) specific primers. EF-1α served as cDNA control. “–” indicates J chain negative control. M (marker) indicates the DNA ladder. The sequences of all PCR products were confirmed by cloning and sequencing.

FISH analysis of gut samples surprisingly showed the presence of J chain^+^ gut epithelial cells (Figure 6A and 6B). Epithelial cells were identified by their size, morphology and apical position in the sample. Cryosections were stained with DAPI to show nuclear morphology and integrity. DAPI staining also allowed identifying epithelial cells characterized by elongated nuclei, located at the basal pole of the cell with an approximate size of one third of the total cell length. The nucleus to cytoplasm ratio of a typical *P. dolloi* gut epithelial cell is shown in Figure S1B, by overlaying the DAPI stain with a DIC image of the same field.

To confirm this result we carried out a RT-PCR experiment using gut epithelial cells obtained by LCM. Pre-pyloric spleen and muscle tissue were used as positive and negative control, respectively. Lungfish spleen expressed all the genes studied: J chain, IgM, IgW and the epithelial cell marker CK-8 (KC579464). This is not surprising as the lungfish pre-pyloric spleen is encased within the first fold of the spiral valve and therefore, our pre-pyloric spleen tissue samples are likely to contain some epithelial cells. Differently, gut epithelial expressed J chain and CK-8 but no B cell markers, indicating that LCM epithelial cells were not contaminated with any possible intraepithelial B cells. This was further confirmed by light microscopy observation of H&E stained cryosections, where no lymphocyte-like cells were found intraepithelially (not shown). Muscle tissue showed no expression of the genes studied (Figure 6C).

## Discussion

The study of key phylogenetic groups like Dipnoi has the potential to reveal important evolutionary processes such as how adaptive immunity evolved with the transition from water to land. However, limited genomic information is available for non-model species such as lungfishes. Furthermore, working with vertebrate species whose genome size is as large as that of Dipnoi (≈100–260 pg), is intrinsically cumbersome. Thus, with the advent of deep sequencing platforms such as 454, gene discovery in non-model species is greatly facilitated, as evidenced in this study.

Joining or “J” chain is a small glycopeptide that, despite not being an immunoglobulin, determines the fate and function of Igs in vertebrates and therefore plays a key role in adaptive immunity [14]. After its initial identification in mammalian species, other J chain sequences have been found in a number of vertebrates [10], [11]. However, to date no J chain had been characterized in sarcopterygian fish. Using 454 deep sequencing of the *P. dolloi* transcriptome we were able to identify the J chain of this species.

Lungfish, coelacanths, and the fish ancestors of the tetrapod lineage all originated within a short time window of about 20 million years, back in the early Devonian (approximately 380 to 400 MYA) [19]. Phylogenetic analyses of the nuclear-encoded recombination activating genes (Rag1 and Rag2), deep sequencing and nuclear gene analysis from all three major lungfish groups, and the Indonesian coelacanth (*Latimeria menadoensis*) DNA sequences, found that the lungfishes represent the closest living relatives of the land vertebrates [19]–[21]. Lungfish display a very unique arrangement of lymphoid tissues, where the majority of the spleen is housed within the first fold of the spiral valve anterior to the ceacum (also called pre-pyloric spleen). The post-pyloric spleen appears as non-encapsulated pieces of lymphoid tissue of variable size located along the central axis of the spiral valve caudal to the pyloric caecum.

African lungfishes possess at least three Ig H chains, IgM, IgW short and IgW long [24]. The previous observation of a 19S high molecular weight antibody in the serum of lungfishes [22], [23] is consistent with the presence of a J chain molecule responsible for the polymerization of Igs. However, the presence and function of a J chain molecule in lungfish had not been reported to date. We initially identified a J chain in our 454-spleen transcriptome database. Cloning of the complete J chain sequence and sequence analysis (Figure 2) revealed important events that have taken place in the evolution of this gene from sarcopterygian fish to mammals. Analysis of the conserved Cys residues present in mammals and in lungfish revealed that six out of eight were conserved and those missing in lungfish form an intramolecular disulphide bond in mammals. Importantly, Cys 2 and 3, which is are most biologically relevant since they are required for the polymerization of Igs and formation of secretory Igs. Both are conserved in lungfish but not in shark or coelacanth. Although still to be proven, it is likely that the function of J chain and its interaction with Igs are both conserved from lungfish to humans. Previous work had identified two key regions within the J chain sequence, named region 2 and region 3, as the most conserved in all tetrapod species [14]. Interestingly, the lungfish but not the coelacanth J chain regions 2 and 3, show high degree of similarity with those of land vertebrates. It is worth noting that region 2 must have diversified considerably in the coelacanth, with the lowest similarity values found across all species analyzed, including the nurse shark. Coelacanth J chain only possesses 4 out of the 8 Cys present in mammals. Importantly, the absence of these Cys as well as the divergence in regions 2 and 3 may indicate that the J chain in this species has different functional role than in other vertebrates. This may indicate that the interactions with putative pIgR in coelacanth may not occur at all or they occur via a different mechanism. Importantly, our phylogenetic analysis and NJ tree (Figure 2C) of the J chain molecule in lungfish and coelacanth supports the idea of lungfishes being the closest living relatives of all tetrapods.

The tissue distribution results (qPCR) indicate that J chain is preferentially expressed in the gut and the post-pyloric spleen of *P. dolloi*. Low levels of transcripts were also detected in the lung, kidney and pre-pyloric spleen. This is in agreement with human J chain expression studies, where the ileum lamina propria and the mesenteric lymph nodes had higher J chain expression levels than the peripheral lymph nodes [3]. Previous work on *X. laevis* and *Rana catesbeiana* also support our results since they found the highest J chain expression levels in the intestine [12]. FISH using a J chain probe found greater number of transcripts in the gut than in the pre-pyloric spleen, supporting the idea that J chain plays an important role in gut immunity. This is in sharp contrast with the nurse shark J chain expression profile, with low levels of J chain expression in the spiral valve (intestine) [11]. Interestingly, despite the blurry anatomical boundaries between mucosal and non-mucosal tissues within Dipnoi, the gut and post-pyloric spleen (both with direct access to the intestinal lumen) express high levels of J chain in lungfish and point to the role of this molecule in mucosal immunity.

The discovery of a J chain molecule in invertebrates [15], where no interaction with Ig molecules is possible, also suggested alternative functional roles for J chain. Interestingly, J chain transcripts were also found in gut epithelial cells by FISH and confirmed by RT-PCR. This is in agreement with the J chain expression pattern found in invertebrates, where cells adjacent to the skin and intestinal surface, were J chain^+^. Authors suggested that the J chain may be involved in the defense system of invertebrate [15] since these cells are in direct contact with foreign environmental substances [15]. In addition, it has been demonstrated that mice express J chain outside the B-cell compartment by a sub-population of dendritic cells [18]. Thus, alternative functions other than Ig polymerization and secretion across epithelial barriers may exist even in higher vertebrates but these results remain controversial today [11], [17], [18]. In this study, we show that J chain is expressed in lungfish mucosal epithelial cells and it is possible that other epithelial cells, not only intestinal, express J chain. Whether the function of J chain in epithelial cells is linked to Igs remains to be elucidated.

Using double staining FISH experiments, we found that a greater proportion (>85%) of gut IgM^+^ cells co-expressed J chain compared to the pre-pyloric spleen (35%). In humans, depending on the methodology employed in each study, the percentage of IgA^+^ J chain^+^ cells can range from 54–74% to 97–100% [4]. This suggests that gut IgM in lungfish has a major role in mucosal secretions and is likely to form multimers. Interestingly, whereas IgM and IgD/IgW are present in sharks, lungfish and teleosts, only shark and lungfish both express IgW and J chain. The multimeric shark IgM is associated with J chain [11], however, it is not known if shark IgW can form complexes with J chain or if shark IgW B cells are capable of co-expressing J chain. Here we report the presence of double IgW^+^Jchain^+^ cells in Dipnoi. We know very little about the presence of IgW and IgM in lungfish mucosal secretions and the roles of SC and J chain in transport across epithelium. In mammals, IgG and IgD B cells also express J chain [4]. Since there is no active transport mechanism for IgG and IgD through human secretory epithelium, J chain expression by these B cell subtypes is thought to be of little significance in mucosal secretions. It is therefore possible that lungfish IgW cells that co-express J chain do not contribute to mucosal immune secretions but have other immune functions. These questions deserve future investigation.

In order to shed light on the importance of lungfish J chain in mucosal immunity we compared the expression of J chain following an experimental oral infection in different lymphoid tissues. J chain transcript expression increased in the gut and, to a lesser extent, the kidney but not in the pre-pyloric spleen of infected individuals compared to controls. Thus, the pathogenic enterobacterium *E. ictaluri* either induces gut B cells to express higher amounts of J chain or results in the recruitment of more B cells in the gut. This is potentially a mechanism of defense against the pathogen since more secretory Igs (in this case IgM and/or IgW) can be transported across the epithelium into the intestinal lumen. Moreover, J chain expression is considered a marker of clonal immaturity [3], implying that the gut and post-pyloric spleen are the sites for the generation of new memory B cell clones, and that terminally differentiated clones populate the lungs, kidney and pre-pyloric spleen. J chain expression also increased following injection with killed *Aeromonas hydrophila* in the Chinese soft shell turtle [37], however, neither the gut nor any other mucosal lymphoid organs were included in the study.

In conclusion, the structural and functional analysis of J chain in sarcopterygian fishes reveals important differences between coelacanths and Dipnoi. Lungfish J chain presents most of the structural canonical features of tetrapod J chains. Thus, generation of polymeric Igs and involvement in mucosal immunity appear as conserved functions of J chain from lungfish to humans. Moreover, our findings support the idea that lungfishes and not coelacanths are the closest relatives to all tetrapods [20], [21]. Importantly, we demonstrated that J chain expression occurs in the gut epithelial cells, IgM and IgW B cells of sarcopterygian fish. The presence of J chain in non-lymphoid cells suggests novel functions for this molecule in Dipnoi.

## Supporting Information

Figure S1 Nucleus to cytoplasm ratio of *P. dolloi* gut lymphocytes (asterisks) (A) and gut epithelial cells (GECs) (B). Cryosections were stained with the nuclear stain DAPI and the fluorescent images (blue) were overlaid with their corresponding differential interference contrast (DIC) image. L: lumen.
(TIF)

File S1 **Supporting tables.**
(DOCX)
