# Supplementary figures and images for: Discovery of J Chain in African Lungfish (Protopterus dolloi, Sarcopterygii) Using High Throughput Transcriptome Sequencing: Implications in Mucosal Immunity
Source: PLoS One. 2013 Aug 14;8(8):e70650. doi: 10.1371/journal.pone.0070650 (PMC3743840; doi:10.1371/journal.pone.0070650)

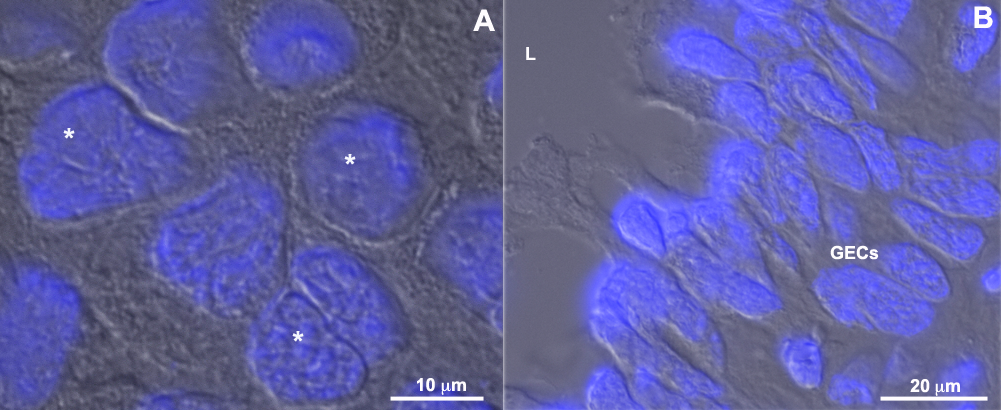

Supplement: Figure S1 — Nucleus to cytoplasm ratio of P. dolloi gut lymphocytes (asterisks) (A) and gut epithelial cells (GECs) (B). Cryosections were stained with the nuclear stain DAPI and the fluorescent images (blue) were overlaid with their corresponding differential interference contrast (DIC) image. L: lumen. (TIF) [file pone.0070650.s001.tif]
