# Supplementary material for: Discovery of J Chain in African Lungfish (Protopterus dolloi, Sarcopterygii) Using High Throughput Transcriptome Sequencing: Implications in Mucosal Immunity
Source: PLoS One. 2013 Aug 14;8(8):e70650. doi: 10.1371/journal.pone.0070650 (PMC3743840; doi:10.1371/journal.pone.0070650)
Supplement: File S1 — Supporting tables. (DOCX) [file pone.0070650.s002.docx]

| **Gene** | **Primer** | **Sequence (5'-3')** | **Application** |
| --- | --- | --- | --- |
|  | 3'Adaptor | CTCGAGATCGATGCGGCCGCTTTTTTTTTTTTTTTTTTTTTT | 3' RACE |
|  | 3'Primer adaptor | CTCGAGATCGATGCGGCCGC | 3' RACE |
| J-Chain | JChainF1 | CCTTCATCTGACACCTGCTAC | 3' RACE |
|  | JChainF2 | CGGAGAAGAAACTGATTCCAGC | 3' RACE |
|  | JChainR1 | CCTTTGCTGGGTCATCAGGAG | 5' RACE |
|  | JChainR2 | CCTGACACAAACACAACTCCTAC | 5' RACE |
|  | JChainR3 | CACACATTTGCACTTTGAATTCACC | 5' RACE |
|  | JChainF0 | CTCCTGATGACCCAGCAAAGG | qPCR & RT-PCR |
|  | JChainR0 | GTAGCAGGTGTCAGATGAAGG | qPCR & RT-PCR |
| IgM | IgMF0 | GCAAACCACTTGTTCCAGGGAG | RT-PCR |
|  | IgMR0 | CATGTAAATTCTTCCTCAGAG | RT-PCR |
|  | IgMF1 | GGTACATTCAGCATCATTAGC | 3' RACE |
|  | IgMdolF2 | GGTCACTATCAAGAAGCACAATGG | 3' RACE |
|  | IgMdolR1 | GGTTCAGAGCATGAAGCAACTGG | 5' RACE |
|  | IgMdolR2 | GACTGCTGCCTATGTATGTTCCC | 5' RACE |
| IgW | IgWF0 | CCTGAAGTGTACAGCAATGGTCG | RT-PCR |
|  | IgWR0 | GTTGAAGTGCCTCTAAACCATGCC | RT-PCR |
|  | IgWdolFall | GACTTAACCTTACGTAATGAAATG | RT-PCR |
|  | IgWdolRall | GCTCAGATCAGTGGGACATTGG | RT-PCR |
| CK-8 | CK-8F0 | GCTGAACTAACCAGGTACATC | RT-PCR |
|  | CK-8R0 | GGTGGCAATTTCAATGTCTAGG | RT-PCR |
| EF-1α | EF1aF | GGAAAGTCTCTCGAGGCAAGG | qPCR & RT-PCR |
|  | EF1aR | CCATACCAGGTTTCAAGACACC | qPCR & RT-PCR |

Table S1: Primers used in this study

| **J chain** | **Region 1** | **Region 2** | **Region 3** | **Overall** |
| --- | --- | --- | --- | --- |
| Mouse | 36.4 | 66.7 | 42.9 | 37.7 |
| Human | 36.4 | 75 | 57.1 | 35.8 |
| Chicken | 45.5 | 66.7 | 57.1 | 44.6 |
| Xenopus | 36.4 | 66.7 | 57.1 | 40.4 |
| Bullfrog | 45.5 | 58.3 | 57.1 | 36.1 |
| Coelacanth | 27.3 | 33.3 | 28.6 | 31.2 |
| Shark | 30.8 | 66.7 | 14.3 | 31.3 |

Table S2. Identity table for J chain sequences (%)
